# Differences in management and outcomes between pheochromocytomas and sympathetic paragangliomas

**DOI:** 10.3389/fendo.2026.1855363

**Published:** 2026-08-19

**Authors:** Mikaela Hermansson Salmi, Jan Calissendorff, Henrik Falhammar

**Affiliations:** 1 Department of Molecular Medicine and Surgery, Karolinska Institute, Stockholm, Sweden; 2 Department of Endocrinology, Karolinska University Hospital, Stockholm, Sweden

**Keywords:** diabetes, hypertension, metastasis, mortality, PPGL, recurrence, treatment

## Abstract

**Purpose:**

The primary aim was to study the differences between pheochromocytomas (PCC) and sympathetic paragangliomas (PGL) in management and short- and long-term outcomes.

**Methods:**

This was a retrospective study of 220 patients that had undergone surgery for PCC or PGL and had been managed during the period 2005–2025 at the Department of Endocrinology, Karolinska University Hospital. Patient data were collected from electronic medical journals.

**Results:**

Patients with PCC compared to PGL had a longer duration of preoperative treatment with alpha-adrenergic receptor antagonist (50.0 vs. 34.0 days, *p* = 0.014) and shorter hospital stay (4.0 vs. 7.0 days, *p* < 0.001). Patients with PCC presented more often with dysglycemia (42.2% vs. 17.6%, *p* = 0.012), and glucose improved to a greater extent as well after surgery (84.2% vs. 33.3%, *p* = 0.033). Patients with PGL were more affected by new related tumors (recurrence, metastases, or metachronous tumor) during the follow-up time (27.8% vs. 11.0%; *p* = 0.019).

**Conclusions:**

There are many similarities between PCC and PGL; however, patients with PGL had a somewhat worse prognosis regarding short- and long-term outcomes, including longer length of stay at the hospital, risk for new associated tumors, and less positive effects on dysglycemia.

## 1 Introduction

Pheochromocytomas (PCCs) and sympathetic paragangliomas (PGLs), collectively known as PPGLs, are rare neuroendocrine tumors with increasing incidence over time, with currently around 0.6 cases per 100,000 per year (1). PCCs are, as of the latest WHO classification, now referred to as adrenal paraganglioma, although in this article the term PCC will be used since this is more widely recognized (2). PPGLs are derived from chromaffin cells and usually release catecholamines, although some are biochemically silent. The adrenal medulla is the primary site of PCCs, while sympathetic PGLs are mostly found along vessels and nerves in the peripheral nervous system (2). PPGLs are often studied as one group because of their shared embryonic origin and similar clinical characteristics related to their ability to release catecholamines, but these tumors can also present heterogenous behavior according to genetic background and tumor size and location. PPGLs have high genetic predisposition, and depending on underlying gene variants, the presentation, management, and outcomes can differ. These variants are categorized into three clusters. Cluster 1 is the pseudohypoxia-related clusters 1A and 1B (e.g., *SDHx*, *VHL*, *FH*, and *EGLN1*). Cluster 2 is the kinase signaling-related cluster (e.g., *RET*, *NF1*, *TMEM127*, and *MAX*). The third cluster is the Wnt signaling-related cluster (e.g., *MAML3*) (3, 4). The PPGLs are no longer classified as benign or malignant since all of these tumors have malignancy potential (2). The only certain way to tell if it is malignant is if metastases are present. The potential risk factors for malignant forms are, e.g., tumor volume and germline variants, especially *SDHB* pathogenic variants (3, 5, 6).

PPGLs can be difficult to diagnose since typical symptoms are also common in numerous other more frequently occurring conditions (7, 8). The most common sign is hypertension, constant or paroxysmal, which is seen in approximately 90% of patients. Symptoms include the classical triad with headache, sweating, and palpitation, but there is a myriad of other vague symptoms like constipation, anxiety, tiredness, nausea, and others (7, 8). Today the majority are found by radiology imaging as incidentalomas, but also the increased screening due to familial syndromes has augmented the prevalence of these tumors (7, 9).

The management of PPGLs depends on various factors, such as the size of the tumor, metastases, preoperative symptoms, and individual health status (6). To manage symptoms and prevent cardiovascular complications such as hypertensive crisis and Takotsubo syndrome, alpha-adrenergic receptor antagonists (α-blockers) such as doxazosin are necessary to use (3, 6, 10, 11). Surgery is the primary choice and considered the only method with curative intention (10). The size of the tumor is included when planning the surgery, where tumors with diameters >6 cm are recommended for open laparotomy and the smaller ones for laparoscopic surgery (6). Laparoscopic surgery has the advantages of faster postoperative recovery and less postoperative complications, while open surgery makes it easier to handle more complex tumor growth (12). Studies have shown that hypertension tends to persist while metabolic conditions, such as diabetes, improve after surgery (13, 14). Metastatic disease occurs in approximately 20% to 25% of PPGLs (15). The recurrence rate range from 5% to 20% (16). There are many similarities between PCCs and PGLs, but also important differences affecting outcomes, even though the data are limited—for example, some studies suggest that PGLs have greater metastatic potential; however, additional studies are needed to confirm this (17, 18).

The objective of the current study was to compare the differences between patients with newly established diagnosis of PCCs and PGLs and candidates for surgical treatments concerning management and short- and long-term outcomes.

## 2 Materials and methods

### 2.1 Patients

The data used in this study were from a retrospective cohort with patients managed at the Department of Endocrinology, Karolinska University Hospital from June 2005 to August 2025, with a total of 233 individuals with PPGLs. However, 13 cases were excluded in this study since they had not undergone surgery; five declined surgery, three died before surgery was performed, two had too extensively metastasized PPGL to undergo curative intended surgery, two had recently been diagnosed and surgery had not yet been performed or data from the surgery was unavailable, and one had only undergone biopsy with removal of the bladder PGL. This resulted in 220 patients meeting the inclusion criteria for being subject for surgery. The cohort was then divided into patients with PCC or PGL.

Data were collected from electronic medical records (EMRs) and included primary diagnosis (PCC/PGL), sex (female/male), age at surgery, largest tumor diameter, gene results, preoperative α-blocker treatment and duration prior to surgery, type of surgery performed (laparoscopic (including robot surgery)/open and if laparoscopic converted into open surgery), length of hospital stay (LOS), follow-up time, cardiovascular events (CVEs) prior to surgery, and perioperative complications (including surgical and anesthesiologic complications and postoperative complications). The biochemical profiles of the cohort and presenting symptoms have been reported previously (23). The number of genes analyzed has expanded over the years, but the latest panel included *EGLN1*, *EPAS1*, *FH*, *MAX*, *MEN1*, *NF1*, *PRKAR1A*, *RET*, *SDHA*, *SDHAF2*, *SDHB*, *SDHC*, *SDHD*, *TMEM127*, and *VHL*. Recurrence, metastases, and new metachronous tumors during follow-up were recorded as well as if the patient was deceased at the time of the last review of the EMR. This was possible because the EMR was linked to the Total Population Register, ensuring that any patient’s death was immediately flagged in the system. Moreover, data on systolic blood pressure (SBP) and diastolic blood pressure (DBP) before and after surgery as well as the number of blood pressure medications prior and after surgery were collected together with data on dysglycemia, including glucose-lowering medications.

Blood pressure improvement was defined as a reduction with at least 10 mmHg when adding together both SBP and DBP and/or with a decreased number of blood pressure medications (and maintaining normotensive pressure). This study recorded the number of blood pressure medications before adding α-blockers preoperatively and the number of blood pressure medications needed after surgery, including α-blockers if it had not been discontinued. Blood pressure medications were only recorded in terms of the number of medications and not in terms of dosages. If both pre- and postoperative blood pressure readings were in the normotensive range (SBP <140 mmHg and DBP <90 mmHg), improvement was still defined as stated above. A reduced number of anti-hypertensive medications but with an increased blood pressure reaching outside of normotensive range was considered to indicate no improvement. Improvement of blood pressure but with added blood pressure medication was not considered an improvement, with the only exception being preoperative SBP ≥180 mmHg and/or DBP ≥120 mmHg with a significant reduction of at least 60 mmHg when adding together SBP and DBP postoperatively and maintained with one blood pressure medication. Type 2 diabetes mellitus (T2DM) was defined as fasting plasma glucose ≥7.0 mmol/L, HbA1c ≥48 mmol/mol, or a random glucose test with plasma glucose >11 mmol/L and when not being considered to be of autoimmune origin. Prediabetic dysglycemia was defined as fasting plasma glucose 6.1–6.9 mmol/L, random plasma-glucose between ≥7.8 and ≤11 mmol/L, or HbA1c ≥42 and <48 mmol/mol at any time. The term dysglycemia encompasses both T2DM and prediabetic dysglycemia. An improvement postoperatively was defined as normalization of HbA1c, disappearance of glycemic disorders, or a reduced dose of the glucose-lowering medications.

Usage of α-blockers prior to surgery was also divided into doxazosin or phenoxybenzamine and their final dose preoperatively. Patients were only included once and with the data from the primary PPGL surgery. Data from additional surgery due to later PPGL recurrence, metastatic disease, or metachronous tumors were included in the follow-up data. Recurrence, new metachronous PPGL, and metastases during follow-up are presented as one group (new associated tumor), and the time in years after primary surgery to first recurrence, metastasis, or metachronous tumor was recorded. Survival was analyzed at the time of the last data collection or at the date of death, whichever occurred earlier. Parts of this cohort have been used previously (7, 19–23).

### 2.2 Statistics

The cohort was divided into patients with PCCs or PGLs and compared. Categorical variables are presented as number (*n*) and proportion (%), continuous variables with non-normal distribution as median and range, and continuous variables with normal distribution as mean and standard deviation (SD). Proportion was calculated by discounting the missing data. Shapiro–Wilks test was used to make sure that the population had normal distribution. If normal distribution was found, Welch’s *t*-test was used; otherwise, it was Mann–Whitney *U*-test. For categorial data, chi-square test with Yates’ continuity correction was used when every cell had frequency ≥5. If cells <5 or if chi-square gave a signal of possible incorrect approximation, Fisher’s exact test was used. Sex distribution was analyzed without Yates’ continuity correction. The Kaplan–Meier method was used to illustrate survival probability, and differences between the groups were analyzed using log-rank test. A *p*-value <0.05 was considered statistically significant for all analyses. Statistical analysis was conducted in R, version 4.5.1 (R Project for Statistical Computing).

## 3 Results

### 3.1 Patients

The majority of the study population was reported to be diagnosed with PCC (*n* = 181, 82.3%) compared to PGL (n = 39, 17.7%). Female sex was overrepresented in the PGL group (69.2%). Median age at surgery was similar between PCC and PGL (57 vs. 51 years, *p* = 0.323). Tumor size was the same in both groups, with a median size of 45 mm [1.58–170]. Preoperative cardiovascular events were observed in 26.5% of patients with PCC and 29.7% of patients with PGL (**Table 1**).

**Table 1:** Baseline characteristics of 220 patients with pheochromocytoma or sympathetic paragangliomas.

Variables	Total PPGL (n = 220)	Pheochromocytomas (n = 181)	Paragangliomas (n = 39)	*P* value
Female (*n*)	120/220 (54.5%)	93/181 (51.4%)	27/39 (69.2%)	0.042
Male (*n*)	100/220 (45.5%)	88/181 (48.6%)	12/39 (30.8%)	0.042
Age at surgery (years)	57 (8–85)	57 (8–85)	51 (18–84)	0.323
Preop CVE (*n*)	59/218 (27.1%)	48/181 (26.5%)	11/37 (29.7%)	0.844
Largest tumor diameter (mm)	45 (1.58–170)	44.5 (5–170)	45 (1.58–170)	0.885
Positive gene panel^a^ (*n*)	40/155 (25.8%)	29/121 (24.0%)	11/34 (32.4%)	0.444

Results are presented as number (*n*) and percentage (%) and group median and range. *P*-values compare PCC and PGL. Bold *P*-values <0.05.

PPGL, pheochromocytomas and paragangliomas; PCC, pheochromocytomas; PGL, paragangliomas; Preop, preoperative; CVE, cardiovascular events.

^a^ PCC tested positive for *RET* (*n* = 13), *NF1* (*n* = 9), *VHL* (*n* = 2), *TMEM127* (*n* = 1), *CAIX* (*n* = 1), *SDHB* (*n* = 1), *MAX* (*n* = 1), and *CACNA1* (*n* = 1). PGL tested positive for *SDHB* (*n* = 9) and *SDHA* (*n* = 2).

A total of 155 patients (70.5%) had undergone gene panel analysis. Positive gene panels were identified in 24.0% of individuals who underwent gene panel analysis: 24.0% of the PCCs and 32.4% of PGLs (*p* = 0.444) (**Table 1**). *RET* variations were most common, followed by *SDHx* and *NF1* variants.

### 3.2 Management

**Table 2** presents the preoperative treatment and surgical data. Treatment with α-blocker for hemodynamic control was used in 203 cases (94.0%). Doxazosin was the most commonly used α-blocker, as noted in 177 of the cases, i.e., 87.2% of all treatment cases used an α-blocker. Phenoxybenzamine was used in 25 cases, and one was treated with intravenous phentolamine, which was included in the phenoxybenzamine group. The median number of treatment days using an α-blocker prior to surgery was longer in patients with PCC than with PGL (50.0 vs. 34.0 days, *p* = 0.014) (**Table 2**).

**Table 2:** Presentation of preoperative treatment and surgical data in patients with pheochromocytomas or sympathetic paragangliomas.

Variables	Total PPGL (*n* = 220)	Pheochromocytomas (*n* = 181)	Paragangliomas (*n* = 39)	*P*-value
Preop α-blocker (*n*)	203/216 (94.0%)	173/179 (96.6%)	30/37 (81.1%)	0.002
Days on preop α-blocker	45 (0–345)	50.0 (0–345)	34.0 (0–143)	0.014
Doxazosin (*n*)	177/214 (82.7%)	150/178 (84.3%)	27/36 (75.0%)	0.271
Phenoxybenzamine^a^ (*n*)	26/214 (12.1%)	24/178 (13.5%)	2/36 (5.6%)	0.265
Final dose doxazosin preop (mg)	25.1 ± 12.6	25.1 ± 12.8	24.9 ± 11.3	0.947
Final dose phenoxybenzamine preop (mg)	68.3 ± 56.2	68.2 ± 58.0	70.0 ± 42.4	0.962
Laparoscopic surgery^b^ (*n*)	146/216 (67.6%)	136/179 (76.0%)	10/37 (27.0%)	<0.001
Laparoscopic surgery even if >6 cm (*n*)	15/51 (29.4%)	15/42 (35.7%)	0/9 (0%)	0.044
Open surgery (*n*)	70/216 (32.4%)	43/179 (24.0%)	27/37 (73.0%)	<0.001
Converted surgery (*n*)	11/146 (7.5%)	8/136 (5.9%)	3/10 (30.0%)	0.029
LOS at hospital (days)	4.0 (2–75)	4.0 (2–75)	7.0 (3–44)	<0.001
Periop complications^c^ (*n*)	42/213 (19.7%)	31/176 (17.6%)	11/37 (29.7%)	0.145

Results are presented as number (*n*) and percentage (%), group median and range, and group mean values and standard deviation (SD). *P*-values compare PCC and PGL. Bold *P*-values <0.05.

PPGL, pheochromocytomas and paragangliomas; PCC, pheochromocytomas; PGL, paraganglioma; Preop, preoperative; α-blocker, alpha-adrenergic receptor antagonist; LOS, length of stay; Periop, perioperative.

^a^ This group includes one treated with phentolamine.

^b^ This group includes surgeries using robot techniques.

^c^ Perioperative complications during hospital admission. Readmitted periods are not included.

Laparoscopic surgery was more commonly used in PCC, while open surgery was the operation method of choice in PGL. Laparoscopic surgeries were less frequently converted to open in the PCC group than in the PGL group (5.9% vs. 30.0%, *p* = 0.029). Laparoscopic approach was employed in 15 patients with PCC where the tumor diameter was greater than 6 cm. No laparoscopic surgery was conducted on PGL tumors greater than 6 cm. Median LOS at hospital was shorter for PCCs compared to PGLs (4.0 vs. 7.0 days, *p* < 0.001). Complications were observed more frequently in the PGL group, but this was not statistically significant (29.7% vs. 17.6%, *p* = 0.145) (**Table 2**).

### 3.3 Short- and long-term outcomes

**Table 3** illustrates the short- and long-term outcomes. Blood pressure before and after surgery did not differ between the PCC and PGL groups. Preoperative hypertension was common in both groups, and normalization of blood pressure was achieved in the majority of both groups after surgery. There was no statistically significant difference between the PCC and PGL groups regarding blood pressure improvement (80.8% vs. 65.6%, *p* = 0.092) or reduction in blood pressure medication (40.3% vs. 25.0%, *p* = 0.148) (**Table 3**).

**Table 3:** Presentation of short- and long-term outcomes in patients with pheochromocytomas or sympathetic paragangliomas.

Variables	Total PPGL (*n* = 220)	Pheochromocytomas (*n* = 181)	Paragangliomas (*n* = 39)	*P*-value
SBP ≥140 mmHg preop (*n*)	142/217 (65.4%)	121/179 (67.6%)	21/38 (55.3%)	0.206
SBP <140 mmHg postop (*n*)	176/217 (81.1%)	146/178 (82.0%)	30/39 (76.9%)	0.609
Preop SBP (mmHg)	149.1 ± 30.3	149.4 ± 29.0	148.1 ± 36.4	0.835
Preop DBP (mmHg)	87.0 ± 16.5	87.2 ± 14.9	86.5 ± 22.9	0.869
Postop SBP (mmHg)	124.8 ± 14.7	124.3 ± 13.9	126.9 ± 18.0	0.401
Postop DBP (mmHg)	75.9 ± 9.7	75.8 ± 9.3	76.1 ± 11.4	0.788
BP improvement postop (*n*)	160/204 (78.4%)	139/172 (80.8%)	21/32 (65.6%)	0.092
Reduced BP medications postop (*n*)	79/208 (40.0%)	71/176 (40.3%)	8/32 (25.0%)	0.148
Dysglycemia preop (*n*)	82/214 (38.3%)	76/180 (42.2%)	6/34 (17.6%)	0.012
Glucose improvement postop (*n*)	66/82 (80.5%)	64/76 (84.2%)	2/6 (33.3%)	0.033
Follow-up time (years)	10.5 ± 9.0	11.0 ± 9.2	8.0 ± 7.5	0.030
New associated tumor^a^ (*n*)	28/199 (14.1%)	18/163 (11.0%)	10/36 (27.8%)	0.019
Time to new associated tumor (years)	6.9 ± 7.7	7.8 ± 8.6	4.3 ± 3.7	0.439
Deceased at last follow-up (*n*)	39/220 (17.7%)	34/181 (18.8%)	5/39 (12.8%)	0.514

Results are presented as number (*n*) and percentage (%) and group mean values and standard deviation (SD). P-values compare PCC and PGL. Bold *P*-values <0.05.

PPGL, pheochromocytomas and paragangliomas; PCC, pheochromocytomas; PGL, paragangliomas; SBP, systolic blood pressure; DBP, diastolic blood pressure; BP, blood pressure; Postop, postoperative; Preop, Preoperative.

^a^ This includes cases with recurrent tumors, metastasis, and new metachronous PPGL tumors.

Prediabetes or T2DM was more commonly noted in patients with PCC than PGL (42.2% vs. 17.6%, *p* = 0.012). Post-surgery improvement was more common in patients with PCC (84.2% vs. 33.3%, *p* = 0.033) (**Table 3**).

Recurrence, metastatic development, or metachronous PPGL tumor was observed less frequently in patients with PCC than in PGL (11.0% vs. 27.8%, *p* = 0.019). No difference in time from primary surgery to detection of new associated tumor was found (7.8 ± 8.6 vs. 4.3 ± 3.7 years, *p* = 0.439) (**Table 3**).

**Figure 1:**
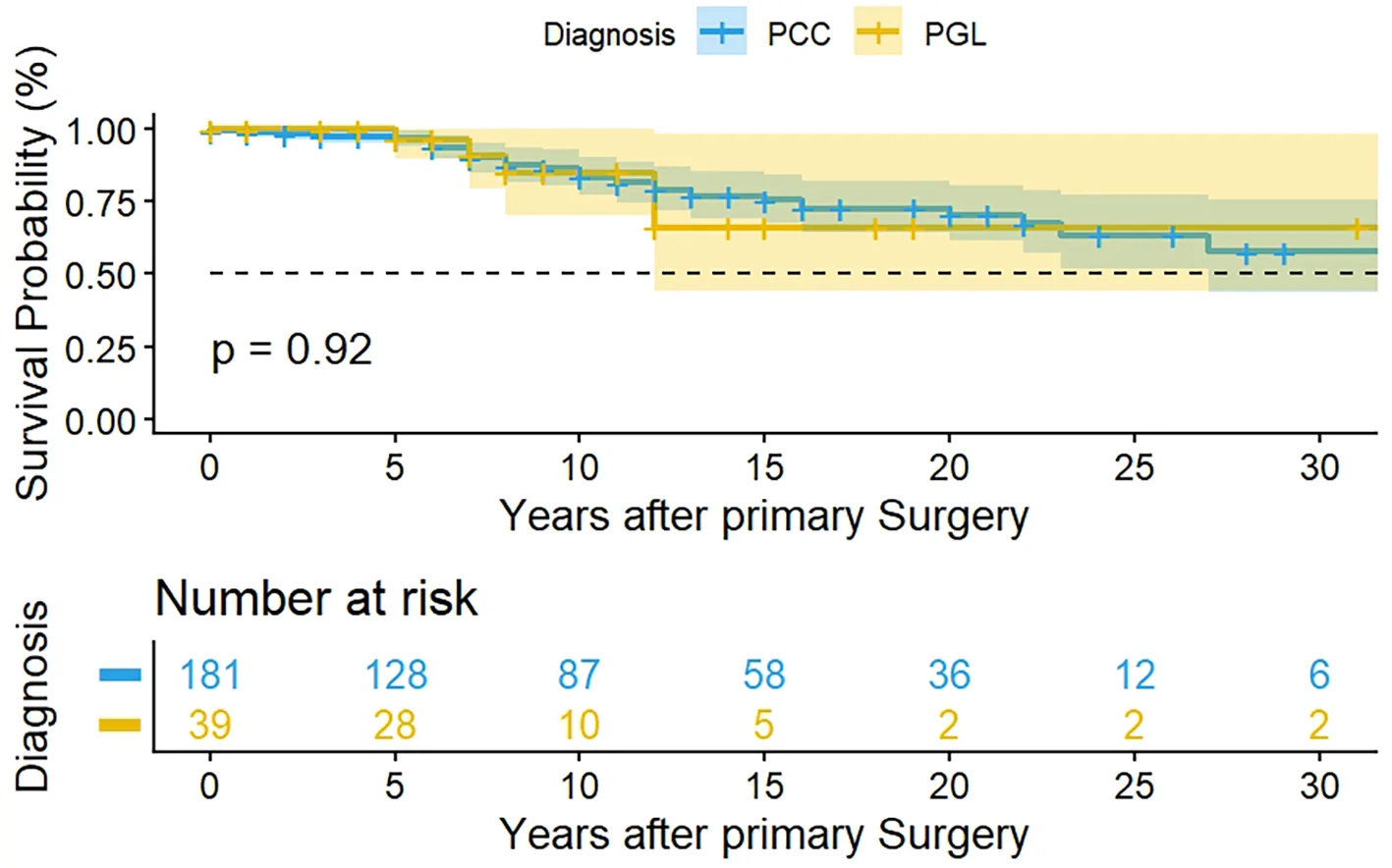
Kaplan–Meier plot for illustration of survival and number at risk during follow-up in patients with pheochromocytomas or sympathetic paragangliomas. Blue indicates PCC and yellow indicates PGL. PCC, pheochromocytomas; PGL, paragangliomas.

No statistical significance was seen upon comparing mortality between the groups (**Table 3**). Survival probability is illustrated using Kaplan–Meier curve as shown in **Figure 1**. Log-rank test showed no statistically significant difference (**Figure 1**).

## 4 Discussion

This is one of the largest cohort studies comparing the differences between PCCs and PGLs concerning management and short- and long-term outcomes. Many similarities between PCCs and PGLs were found, but patients with PGLs had worse outcomes regarding postoperative LOS, frequency of new PPGL-related tumors, and effects on dysglycemia. In spite of often presenting similar clinical characteristics, PCC and PGL exhibit numerous differences and thus may need to be considered and investigated depending on the factors causing their heterogenous behavior rather than the shared embryonic origin.

As expected, open surgery was more commonly used in patients with PGL in accordance with current guidelines recommending open surgery for PGLs with large or invasive tumors and depending on anatomical localization (6). More laparoscopic surgeries were converted to open in patients with PGL than PCC. No statistical significance was seen in perioperative complications. However, LOS was longer for patients with PGL. Prolonged hospital stay after adrenalectomy has been seen in open surgery and converted laparoscopic surgeries in patients with PCC (24). Tumor size >5.6 cm has also been suggested as an individual risk factor for prolonged hospital stay in patients with PPGLs (25). Current management guidelines recommend open surgery for PPGL tumors >6 cm (6), while a retrospective study of 65 patients with PCCs imply that laparoscopic approaches for tumors >6 cm do not carry higher risks for complications and malignancy development (26). In our study, 15 cases with tumor size >6 cm had been subjects for laparoscopic surgical approach. Tumor complexity and site are the other factors considered when choosing the surgical method, with tumor involving multiple organs and systems making it more complicated and risk for vascular injuries being a threat, and this is more often seen in patients with PGL (27). The prolonged LOS for patients with PGL in this study most likely reflects the anatomical location of tumors and the more commonly used method of open surgery in this group rather than tumor size and intrinsic differences between PCCs and PGLs. With the emerging minimal invasive surgical techniques and their advantages, one can speculate that more PPGL surgeries will be conducted using these in the future (28). One investigation found that robot surgery could be a suitable choice even for larger PPGL tumors (29). Thus, it is important to have individualized surgical strategies and multidisciplinary team evaluation for individual cases (27).

Preoperative treatment with α-blockers is believed to better control the hemodynamic situation and decrease the perioperative morbidity rate, with less hypertensive crises (6, 11). An interesting observation in our study was that patients with PGL had a shorter duration of treatment with α-blockers prior to surgery compared to patients with PCC. Current guidelines conclude that 7–14 days only of α-blocker treatment before surgery is enough (6). However, many centers use longer preoperative α-blockage, e.g., a study from Poland showed a median time of 44 days for doxazosin, which is very similar to our median time of 45 days (30). One smaller study (*n* = 108) found no differences in dosages and length of treatment between PCC and PGL (20). Yao et al. discussed the effects of different secretory phenotypes where epinephrine-releasing tumors are connected to more intraoperative hemodynamic instability (31). Since sympathetic PGL almost exclusively release norepinephrine, this could explain the shorter need for preoperative α-blockage (23).

### 4.1 Hypertension

Hypertension is known to be the singular most common sign in patients with PPGL (32). Studies have shown that persistent hypertension ranges between 7% to 58% in patients that have undergone resection for PCC (33). A recent study by Lu et al. of 259 patients with PCC found persistent hypertension in 40.9%. The authors listed older age, duration of preoperative hypertension, and previous cardiovascular events as potential risk factors to persistent hypertension post-surgery (34). They also conclude that a larger tumor size was a factor in their univariable analysis, but it was not a statistically relevant factor in their multivariable analysis. An earlier study indicated age as a risk factor for persistent hypertension postoperatively but negated sex as one (35). In our study, there was no statistical difference in comparing improvement in blood pressure postoperatively in patients with PCC and PGL. The median age at diagnosis and surgery, tumor size, and the frequency of preoperative cardiovascular events were similar in the PCC and PGL groups, which may explain the comparable effects on hypertension. These results call for more studies on the subject that takes confounders into account.

### 4.2 T2DM and prediabetes

Our study found that 38.3% had preoperative glycemic abnormalities, including T2DM and prediabetes, and this was almost 2.4 times more common in patients with PCC than in PGL. A study of glycemic abnormalities in patients with PCC showed a prevalence of 49.5% and remission in approximately 60% of the patients post-surgery (36). Another investigation observed glucose abnormalities in 35.4% of their cohort, consisting of both PCC and sympathetic PGLs, and remission in more than 50% (37). Our data suggest a higher number of glycemic improvements, seen in 80.5% of the total cohort but only in 33% of patients with PGLs. A recent large study found that 70% had manifest glucose disorders and found key risk factors being age at diagnosis and norepinephrine concentrations (38). A possible explanation could be that PGL usually secretes norepinephrine, while epinephrine mainly is produced by PCC. Epinephrine has a larger impact on glucose and insulin secretion, while norepinephrine contributes to increased insulin resistance (23, 39). However, there is a very limited number of studies directly comparing the difference in prevalence of dysglycemia between PCCs and PGLs (23).

### 4.3 New related tumors and mortality

A higher rate of new PPGL-associated tumors was found in the PGL group compared to the PCC group in our cohort, which is consistent with a previous study (17). Patients with PGLs more often presented with positive gene panels; however, this was not statistically different in our study, which is likely due to power issues. *SDHx* variants have been associated with a more aggressive disease (3, 40). In our study, all patients with PGL and positive gene panels had *SDHx* variants, and 82% had a *SDHB* variant. However, long-term overall survival in our PCC and PGL groups was similar. Long-term follow-up in patients with PPGL is required, even in tumors without a known gene variant, since metastases can occur after several years (5). The mean time for a new associated tumor event of recurrence, metastases, or metachronous tumors after primary surgery was 6.9 years in our study, with some tumors noted after decades of follow-up.

### 4.4 Strengths and limitations

This study is one of the largest studies comparing patients with PCCs and PGLs concerning management and outcomes. Many details were available and analyzed. However, despite being one of the largest studies, the limited number, especially in the PGL group, affected the possibility of finding smaller differences. Moreover, a retrospective study has its limitations such as potential biases and missing data.

Confounders linked to clinical variability were reduced using a single-center cohort design. This minimizes the risk of heterogenous quality, with guidelines and definitions being the same. However, the gathering of data stretches over 20 years, and clinical routines may have changed over time. Mainly, this is seen in our results for genetic testing, with increased testing in later years. Similarly, the measurements of urinary/plasma catecholamines/metanephrines have changed over the years. This prevented us from classifying the cohort according to adrenergic/noradrenergic biochemical phenotype as suggested by Eisenhofer et al. (41). The large time span of data collection also results in considerable variability in the duration of follow-up among patients. This heterogeneity may have influenced the assessment of long-term outcomes and overall mortality. Hospital-specific expertise and resource availability may influence outcome measures such as complication rates or access to follow-up care, limiting the ability to extrapolate the findings to a broader population. Nevertheless, mortality was verified through the National Population Register, which captures all deaths occurring in Sweden.

As the data were gathered manually, some degree of information bias is possible. In this case, data were collected by two of the authors (JC and HF), which reduces the risk of systematic bias but also increases the risk of interobserver variability. For some patients, data points were missing, which could lead to skewness in the results, especially in the PGL group due to its limited size.

### 4.5 Conclusion

A lot of similarities between patients with PCCs and patients with PGLs were found. However, some important differences in preoperative treatment, type of surgery, and short- and long-term outcomes were present. Patients with PGLs had somewhat worse prognosis with longer LOS, higher frequency of new PPGL-associated tumors, and less positive effects on dysglycemia after surgery. More studies comparing surgical methods, secretory phenotypes, and outcomes are needed to further improve the management of these tumors and short- and long-term outcomes.

## Data Availability

The datasets presented in this article are not readily available because of privacy and legal reasons. Requests to access the datasets should be directed to Henrik Falhammar, henrik.falhammar@ki.se.

## References

[1] Al Subhi AR Boyle V Elston MS . Systematic review: incidence of pheochromocytoma and paraganglioma over 70 years. J Endocr Soc. (2022) 6:bvac105. doi: 10.1210/jendso/bvac105 35919261 PMC9334688

[2] Mete O Asa SL Gill AJ Kimura N de Krijger RR Tischler A . Overview of the 2022 WHO classification of paragangliomas and pheochromocytomas. Endocr Pathol. (2022) 33:90–114. doi: 10.1007/s12022-022-09704-6 35285002

[3] Calissendorff J Juhlin CC Bancos I Falhammar H . Pheochromocytomas and abdominal paragangliomas: a practical guidance. Cancers. (2022) 14:917. doi: 10.3390/cancers14040917 35205664 PMC8869962

[4] Nölting S Bechmann N Taieb D Beuschlein F Fassnacht M Kroiss M . Personalized management of pheochromocytoma and paraganglioma. Endocr Rev. (2021) 43:199–239. doi: 10.1210/endrev/bnab019 34147030 PMC8905338

[5] Granberg D Juhlin CC Falhammar H . Metastatic pheochromocytomas and abdominal paragangliomas. J Clin Endocrinol Metab. (2021) 106:e1937–e1952. doi: 10.1210/clinem/dgaa982 33462603 PMC8063253

[6] Lenders JWM Duh Q-Y Eisenhofer G Gimenez-Roqueplo A-P Grebe SKG Murad MH . Pheochromocytoma and paraganglioma: an endocrine society clinical practice guideline. J Clin Endocrinol Metab. (2014) 99:1915–42. doi: 10.1210/jc.2014-1498 24893135

[7] Falhammar H Kjellman M Calissendorff J . Initial clinical presentation and spectrum of pheochromocytoma: a study of 94 cases from a single center. Endocr Connect. (2018) 7:186–92. doi: 10.1530/EC-17-0321 29217652 PMC5776668

[8] Geroula A Deutschbein T Langton K Masjkur J Pamporaki C Peitzsch M . Pheochromocytoma and paraganglioma: clinical feature-based disease probability in relation to catecholamine biochemistry and reason for disease suspicion. Eur J Endocrinol. (2019) 181:409–20. doi: 10.1530/EJE-19-0159 31370000

[9] Berends AMA Lenders JWM Kerstens MN . Update on clinical characteristics in the evaluation of phaeochromocytoma and paraganglioma. Best Pract Res Clin Endocrinol Metab. (2024) 38:101953. doi: 10.1016/j.beem.2024.101953 39384447

[10] Neumann HPH Young WF Eng C . Pheochromocytoma and paraganglioma. N Engl J Med. (2019) 381:552–65. doi: 10.1056/NEJMra1806651 31390501

[11] Y-Hassan S Falhammar H . Cardiovascular manifestations and complications of pheochromocytomas and paragangliomas. J Clin Med. (2020) 9:2435. doi: 10.3390/jcm9082435 32751501 PMC7465968

[12] Zhou Y Tai Y Shang J . Progress in treatment and follow-up of pheochromocytoma. Eur J Surg Oncol. (2025) 51(8):110144. doi: 10.1016/j.ejso.2025.110144 40373734

[13] Bentebbaa FZ Rami I Assarrar I Elamel R Boutaybi L Rouf S . Evolution of metabolic disorders after resection of pheochromocytomas and paragangliomas: a single-center study. Med Pharm Rep. (2025) 98:190–5. doi: 10.15386/mpr-2741 40371414 PMC12070968

[14] Jiang X Guo N Zhang H Wang C Jing H Liu T . Long-term persistent hypertension following surgical resection of pheochromocytoma and paraganglioma. Endocr Connect. (2026) 15:e250714. doi: 10.1530/EC-25-0714 41693540 PMC12989711

[15] Varghese J Skefos CM Jimenez C . Metastatic pheochromocytoma and paraganglioma: integrating tumor biology in clinical practice. Mol Cell Endocrinol. (2024) 592:112344. doi: 10.1016/j.mce.2024.112344 39182716

[16] Araujo-Castro M García Sanz I Mínguez Ojeda C Hanzu F Mora M Vicente A . Local recurrence and metastatic disease in pheochromocytomas and sympathetic paragangliomas. Front Endocrinol. (2023) 14:1279828. doi: 10.3389/fendo.2023.1279828 38155946 PMC10753179

[17] Ayala-Ramirez M Feng L Johnson MM Ejaz S Habra MA Rich T . Clinical risk factors for Malignancy and overall survival in patients with pheochromocytomas and sympathetic paragangliomas: primary tumor size and primary tumor location as prognostic indicators. J Clin Endocrinol Metab. (2011) 96:717–25. doi: 10.1210/jc.2010-1946 21190975

[18] Iguchi DYV Martins Filho SN Soares IC Siqueira SAC Alves VAF Assato AK . Identification of predictors of metastatic potential in paragangliomas to develop a prognostic score (PSPGL). J Endocr Soc. (2024) 8:bvae093. doi: 10.1210/jendso/bvae093 38799767 PMC11112433

[19] Dahl MAJ Calissendorff J Falhammar H . Sex differences in management and outcomes in pheochromocytomas and paragangliomas. Front Endocrinol. (2025) 16:1597908. doi: 10.3389/fendo.2025.1597908 40589516 PMC12206822

[20] Falhammar H Kjellman M Calissendorff J . Treatment and outcomes in pheochromocytomas and paragangliomas: a study of 110 cases from a single center. Endocrine. (2018) 62:566–75. doi: 10.1007/s12020-018-1734-x 30220006 PMC6244895

[21] Falhammar H Stenman A Calissendorff J Juhlin CC . Presentation, treatment, histology, and outcomes in adrenal medullary hyperplasia compared with pheochromocytoma. J Endocr Soc. (2019) 3:1518–30. doi: 10.1210/js.2019-00200 31384714 PMC6676072

[22] Ali NA Calissendorff J Falhammar H . Sex differences in presentation of pheochromocytoma and paraganglioma. Front Endocrinol. (2025) 16:1463945. doi: 10.3389/fendo.2025.1463945 39917539 PMC11798811

[23] Pihlblad VED Calissendorff J Falhammar H . Differences in clinical presentation between pheochromocytomas and paragangliomas. Clin Endocrinol (Oxf). (2025) 103:651–8. doi: 10.1111/cen.70002 40641054 PMC12492778

[24] Parente A Kamarajah SK Thompson JP Crook C Aspinall S Melvin R . Risk factors for postoperative complications after adrenalectomy for phaeochromocytoma: multicentre cohort study. BJS Open. (2023) 7:zrad090. doi: 10.1093/bjsopen/zrad090 37757753 PMC10533033

[25] Ma L Yu X Huang Y . Risk factors for postoperative complications after pheochromocytoma and/or paraganglioma: a single-center retrospective study. Front Oncol. (2023) 13:1174836. doi: 10.3389/fonc.2023.1174836 37213287 PMC10198611

[26] Wilhelm SM Prinz RA Barbu AM Onders RP Solorzano CC . Analysis of large versus small pheochromocytomas: operative approaches and patient outcomes. Surgery. (2006) 140:553–9. doi: 10.1016/j.surg.2006.07.008 17011902

[27] Wang X Zhao Y Liao Z Zhang Y . Surgical strategies of complicated pheochromocytomas/paragangliomas and literature review. Front Endocrinol. (2023) 14:1129622. doi: 10.3389/fendo.2023.1129622 37152961 PMC10160616

[28] Fang AM Rosen J Saidian A Bae S Tanno FY Chambo JL . Perioperative outcomes of laparoscopic, robotic, and open approaches to pheochromocytoma. J Robot Surg. (2020) 14:849–54. doi: 10.1007/s11701-020-01056-9 32112185 PMC8983095

[29] He J Li Y Han K Su S Wang J Wang W . Retrospective analysis of robotic versus laparoscopic surgery in the treatment of giant pheochromocytoma and paraganglioma. J Robot Surg. (2025) 19:206. doi: 10.1007/s11701-025-02371-9 40335861

[30] Zawadzka K Pisarska-Adamczyk M Hubalewska-Dydejczyk A Pędziwiatr M . Prolonged alpha-blockade and doxazosin are associated with hypertensive crisis in pheochromocytoma surgery. Front Endocrinol. (2026) 16. doi: 10.3389/fendo.2025.1682912 41573200 PMC12819246

[31] Yao Y Guo Y Fan J Lin T Wang L Zhang S . Influence of duration of preoperative treatment with phenoxybenzamine and secretory phenotypes on perioperative hemodynamics and postoperative outcomes in pheochromocytoma and paraganglioma. Front Endocrinol. (2023) 14:1139015. doi: 10.3389/fendo.2023.1139015 37152936 PMC10154584

[32] Yu Y Chen C Meng L Han W Zhang Y Zhang Z . Hypertension and cardiac damage in pheochromocytoma and paraganglioma patients: a large-scale single-center cohort study. BMC Cardiovasc Disord. (2024) 24:325. doi: 10.1186/s12872-024-03936-6 38926862 PMC11200840

[33] Prakash P Ramachandran R Tandon N Kumar R . Changes in blood pressure, blood sugar, and quality of life in patients undergoing pheochromocytoma surgery: a prospective cohort study. Indian J Urol IJU J Urol Soc India. (2019) 35:34–40. doi: 10.4103/iju.IJU_190_18 30692722 PMC6334590

[34] Lu S Wang C Tian X Zhang T . Identification of risk factors for postoperative persistent hypertension in patients with pheochromocytoma. J Surg Oncol. (2024) 130:380–5. doi: 10.1002/jso.27764 39082432

[35] Plouin PF Chatellier G Fofol I Corvol P . Tumor recurrence and hypertension persistence after successful pheochromocytoma operation. Hypertens Dallas Tex 1979. (1997) 29:1133–9. doi: 10.1161/01.hyp.29.5.1133 9149678

[36] Elenkova A Matrozova J Vasilev V Robeva R Zacharieva S . Prevalence and progression of carbohydrate disorders in patients with pheochromocytoma/paraganglioma: retrospective single-center study. Ann Endocrinol. (2020) 81:3–10. doi: 10.1016/j.ando.2020.01.001 32067697

[37] Araujo-Castro M Mínguez Ojeda C García Centeno R López-García M-C Lamas C Hanzu FA . Glycemic disorders in patients with pheochromocytomas and sympathetic paragangliomas. Endocr Relat Cancer. (2022) 29:645–55. doi: 10.1530/ERC-22-0218 36069783

[38] Zhang W Yu J Chen Y Zhou Y Cui Y Li T . Glucose disorders in patients with pheochromocytoma/paraganglioma: profile and influence effects in a large cohort with 705 patients. Endocr Pract Off J Am Coll Endocrinol Am Assoc Clin Endocrinol. (2025) 31:269–77. doi: 10.1016/j.eprac.2024.11.004 39551188

[39] Abe I Islam F Lam A-Y . Glucose intolerance on phaeochromocytoma and paraganglioma-the current understanding and clinical perspectives. Front Endocrinol. (2020) 11:593780. doi: 10.3389/fendo.2020.593780 33324347 PMC7726412

[40] Kimura N Takekoshi K Naruse M . Risk stratification on pheochromocytoma and paraganglioma from laboratory and clinical medicine. J Clin Med. (2018) 7:242. doi: 10.3390/jcm7090242 30150569 PMC6162838

[41] Eisenhofer G Pamporaki C Lenders JWM . Biochemical assessment of pheochromocytoma and paraganglioma. Endocr Rev. (2023) 44:862–909. doi: 10.1210/endrev/bnad011 36996131

